# The Single-Parameter Bragg–Williams Model for Eutectic Solvents

**DOI:** 10.3390/ijms26030997

**Published:** 2025-01-24

**Authors:** Ozge Ozkilinc, Miguel Angel Soler, Paolo Giannozzi, Santiago Aparicio, Federico Fogolari

**Affiliations:** 1 Dipartimento di Scienze Matematiche, Informatiche e Fisiche (DMIF), University of Udine, 33100 Udine, Italy; ozkilinc.ozge@spes.uniud.it (O.O.); miguelangel.solerbastida@uniud.it (M.A.S.); paolo.giannozzi@uniud.it (P.G.); 2 Department of Chemistry, University of Burgos, 09001 Burgos, Spain; sapar@ubu.es; 3 International Research Center in Critical Raw Materials for Advanced Industrial Technologies (ICCRAM), University of Burgos, 09001 Burgos, Spain

**Keywords:** Bragg–Williams, eutectic solvents, binary mixtures

## Abstract

The study of solid–liquid equilibria offers critical insights into the molecular interactions between constituents in binary mixtures. Predicting these equilibria often requires comprehensive thermodynamic models, yet simplified approaches can provide valuable perspectives. In this work, we explore the application of the Bragg–Williams model to solid–liquid equilibria in binary mixtures leading to the formation of eutectic solvents. This model relies on a single parameter—the molar energy change upon mixing compounds—and demonstrates noteworthy features: the parameter can be estimated from a few (in principle, from a single) experimental melting points, and it correlates strongly with interaction energy parameters from more complex models, such as the PC-SAFT molecular-based equation of state. By using the Bragg–Williams model, we provide a straightforward and informative framework for characterizing solid–liquid equilibria, enabling insights into molecular interactions while requiring few data points as input. Despite its simplicity, the model effectively captures the essence of binary mixture energetics, positioning it as a practical tool for advancing the understanding of phase behavior in eutectic solvent systems.

## 1. Introduction

In the last 20 years, environmental concerns have led to the development of a class of more eco-friendly solvents that have been termed deep eutectic solvents (DESs) due to the large depression in the melting temperature compared to the melting temperature of pure compounds [1,2]. Eutectic solvents have been proposed for several technological applications such as green solvents, electrochemistry, batteries, gas separation operations, catalysis, biomass processing, and pharmaceutical or nanomaterial processing, among others [3].

For immiscible solids, the lowering of the melting point in ideal mixtures is entirely due to the mixing entropy contribution favoring the liquid versus the solid state.

In fact, non-ionic eutectic mixtures, with eutectic melting temperatures lower than ideal solutions, have been known for a long time (e.g., the menthol/camphor and menthol/thymol eutectic properties are reported in a medical book in 1894 [4]).

Deviations from ideality have been linked to differences in interactions in the mixture and in the pure compounds, where more favorable interactions in the mixture lead to a lowering of the melting point and, conversely, less favorable interactions in the mixture lead to an increase of the melting point. The modeling of melting temperatures and many other properties of binary mixtures involves different approaches and has descriptive or predictive purposes.

### 1.1. Theoretical Models of Solid–Liquid Equilibria

The modeling of the thermodynamic properties of eutectic mixtures has been the subject of recent reviews [5,6]. Many approaches are based on empirical equations for each property to be fitted to experimental data. When large quantities of data are available, a given equation form may be fitted to molecular descriptors, like in Quantitative Structure Activity Relationship methods [7], or learned by machine learning algorithms [8].

Theoretical models to describe solid–liquid equilibria have been developed based on parametric thermodynamic modeling [9,10], group contribution [11], conductor-like screening model for real solvents (COSMO-RS) [12], and machine learning models [8].

From the physicochemical point of view, approaches based on classical statistical mechanics are more interesting compared to those based on extensive parametrization, because, even using fitted parameters, it is possible to link such parameters to molecular properties.

Equation of state models use pure compounds’ quantities and specific pairwise (empirical or predicted) parameters to establish a relation among pressure, temperature, and volume, from which several properties, e.g., compressibility and speed of sound, are derived.

Phase equilibria are typically modeled by writing an expression for the activity coefficient or the excess Gibbs’ free energy. The non-random two-liquid (NRTL) model does so by using a limited number of parameters [13]. The latter may be fitted to experimental data or predicted by theoretical methods.

Other approaches, such as perturbed chain statistical associating fluid theory (PC-SAFT) [14,15], model thermodynamic quantities of the solution using pure compound molecular properties, which are specifically combined for the mixture, and interaction terms specific for each binary mixture. PC-SAFT achieves great predictivity by using also a limited number of parameters. For this reason, it has been widely used in modeling eutectic systems.

The reader is referred to the cited reviews for a comprehensive list of approaches and specific references.

In the following, we explore a statistical mechanics-based model, the Bragg–Williams model, which is by far simpler than all the methods mentioned above, and condenses information on molecular interactions in the eutectic mixture with respect to interactions in the pure compounds into a single energetic parameter, as discussed later, which allows us to model phase equilibria for binary mixtures.

### 1.2. Applications of the Bragg–Williams Models to Eutectic Mixtures

The Bragg–Williams model (also referred to as the Bragg–Williams approximation) has been formulated to study the effect of thermal agitation on atomic arrangement in alloys in the 1930s [16,17,18] and it has been used mainly in the same context, but also in other contexts, since then. We will follow here the description of the model given by T. L. Hill [19].

The application of the model to the study of eutectic binary mixtures has not been widely pursued. McCammon and Deutch first used the model to describe phase transitions in a phospholipid bilayer [20] and Lee [21] reports the equation for the solid–liquid equilibrium temperature in a review on phase diagrams and phase transitions of lipid mixtures. The derivation is not made explicit but refers to the standard derivation for ideal solutions. Later, the same model was applied by Dorset [22] to study the eutectic properties of binary mixtures of cholesterol, cholesteryl esters, and triacylglycerols.

Other studies focused on applying, evaluating, and extending the Bragg–Williams approximation to eutectic systems. Experimentally driven research has directly assessed the Bragg–Williams approximation for binary mixtures. For example, Joshi et al. [23] applied the model to binary fat mixtures, such as cocoa butter/cocoa oil, to model phase diagrams and calculate interaction parameters (χ in their paper) relevant to the model. The study highlights the model’s effectiveness in identifying non-ideal mixing behavior while pointing out its inability to capture polymorphism in fat blends. Similarly, Macridachis-Gonzalez et al. [24] used the model to analyze binary triacylglycerol (TAG) mixtures, focusing on immiscibility and eutectic properties due to TAG–TAG interactions, but also noting the model’s limitations in fully describing solid-state behavior. A theoretical and computational application to adsorption phenomena has been presented by Davila et al. [25].

Here, we present a detailed derivation of the Bragg–Williams model; we apply the model to eutectic and deep eutectic solvents showing its effectiveness; and we discuss the dependence of the model on uncertainties in the mixture melting temperature and enthalpy and temperature of melting of pure compounds.

We show that straightforward application of the model to deep eutectic solvents, though lacking the flexibility of other much more refined and complex approaches, enables one to find a useful energy parameter, the only parameter used by the model, that has a direct physical interpretation.

## 2. Results and Discussion

### 2.1. Fitting Solid–Liquid Equilibrium Curves with the Bragg–Williams Model

The capability of a single-parameter model like the Bragg–Williams model to reproduce the solid–liquid equilibria of deep eutectic mixtures has been tested on a series of type V DESs, for which data were obtained by Coutinho and collaborators [26] based on the combination of one of two terpenes (menthol and thymol) with one of six monocarboxylic acids (capric acid, caprylic acid, lauric acid, myristic acid, palmitic acid, and stearic acid). In this paper, the solid–liquid equilibrium curves were fitted by PC-SAFT, which also provides predictions of other physicochemical properties. Type V DESs were preferred over other DESs because, being non-ionic, they are better described by the Bragg–Williams model, which does not take into account salt dissociation and long-range electrostatic effects. Moreover, this class of DES is of interest for technological applications because it is natural and non-toxic, has lower viscosity, and is suitable for flexible design [27].

**Figure 1:**
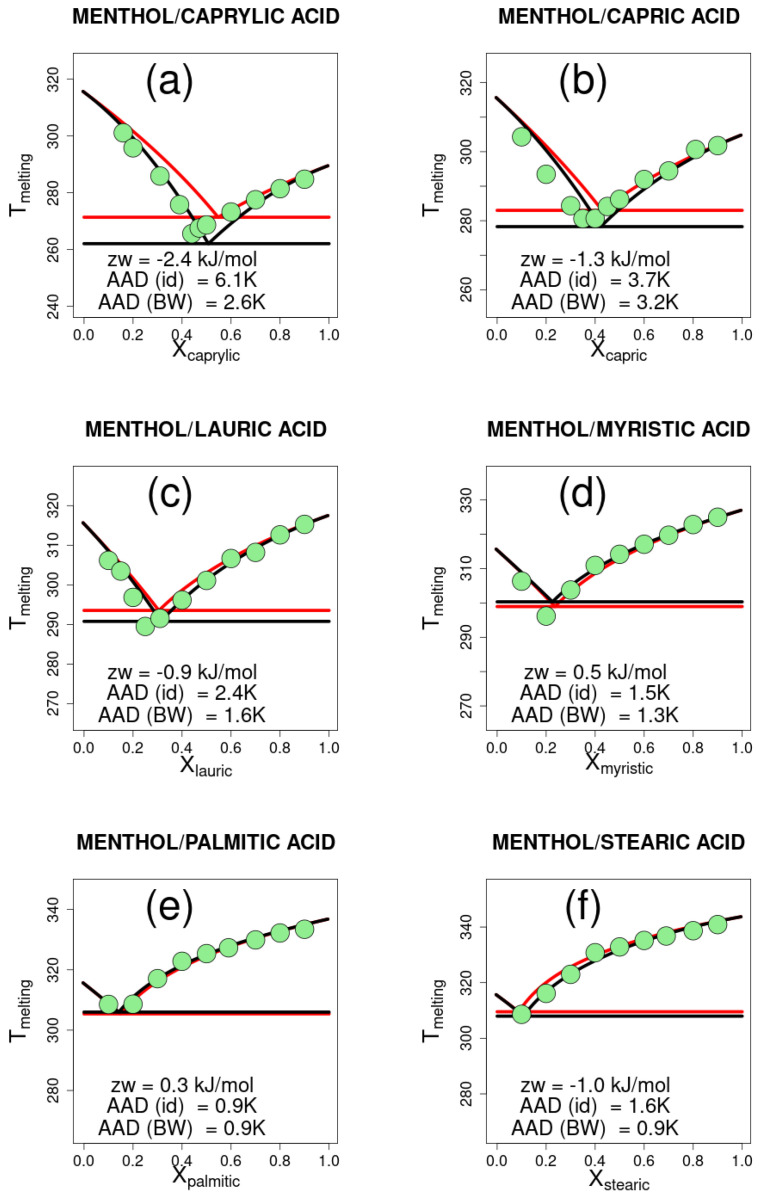
Solid–liquid equilibrium for eutectic mixtures of menthol with six different monocarboxylic acids. Experimental points are shown as green filled circles for menthol mixtures with (**a**) caprylic acid, (**b**) capric acid, (**c**) lauric acid, (**d**) myristic acid, (**e**) palmitic acid, and (**f**) stearic acid. The solid–liquid equilibrium curves are drawn for ideal mixtures (red line) and the Bragg–Williams model (black line). Horizontal lines are drawn at the lowest melting temperature. The corresponding best-fit Bragg–Williams model zw parameter and the absolute average deviation (AAD) for the ideal (id) and Bragg–Williams (BW) models are reported in the plot.

**Figure 2:**
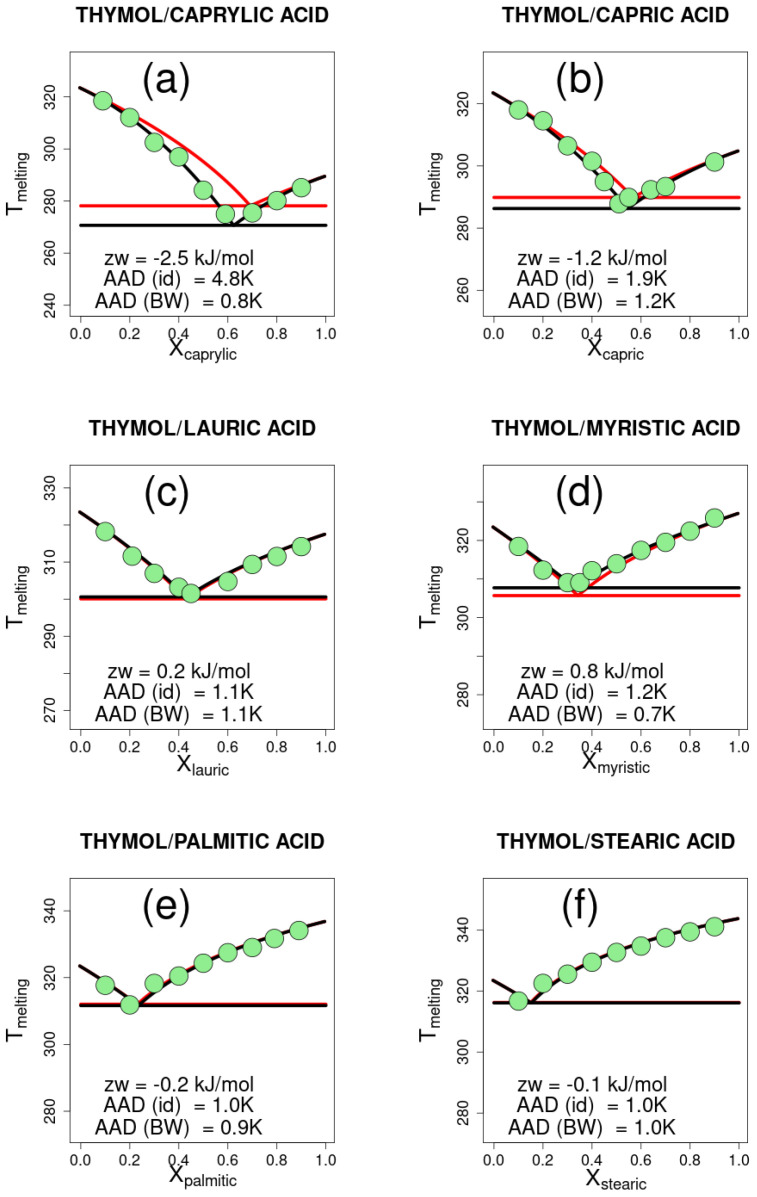
Solid–liquid equilibrium for eutectic mixtures of thymol with six different monocarboxylic acids. Experimental points are shown as green filled circles for thymol mixtures with (**a**) caprylic acid, (**b**) capric acid, (**c**) lauric acid, (**d**) myristic acid, (**e**) palmitic acid, and (**f**) stearic acid. The solid–liquid equilibrium curves are drawn for ideal mixtures (red line) and the Bragg–Williams model (black line). Horizontal lines are drawn at the lowest melting temperature. The corresponding best-fit Bragg–Williams model zw parameter and the absolute average deviation (AAD) for the ideal (id) and Bragg–Williams (BW) models are reported in the plot.

The experimental data are shown in Figure 1 and Figure 2, together with the curves drawn for the ideal mixtures and for the Bragg–Williams model. For each mixture, the melting temperature values of the pure compounds and the enthalpy of fusion are used to estimate the parameter zw. In practice, zw is varied from −100 to 100 kJ in steps of 0.1 kJ/mol, and the melting temperature is calculated according to Equation (21) for one thousand equally spaced values of $X_{A}$ between 0 and 1. For each molar composition, the highest of the melting temperatures computed for substances *A* and *B* is taken as the solid–liquid equilibrium temperature.

The average absolute deviation (AAD) between the calculated and experimental data ranges from 0.7 to 3.2 K, and is on average 1.35 ± 0.78 K. For the same data, the AAD of the PC-SAFT model is 1.15 K, which is just 0.20 K lower. The AAD is in some cases large, but in all cases, it is less than the AAD for the ideal mixture, which ranges from 0.9 to 6.1 K, being on average 2.26 ± 1.71 K.

The calculated values zw represent the change in energy upon forming *z* contacts in the mixture by breaking $\frac{z}{2}$ contacts in each of the pure liquids. Depressions in the eutectic point, compared to ideal solutions, are associated with negative values of zw, whereas systems that behave similarly to ideal mixtures have a zw close to zero. Positive values of zw are associated with an increase in the melting temperature with respect to ideal mixtures.

**Figure 3:**
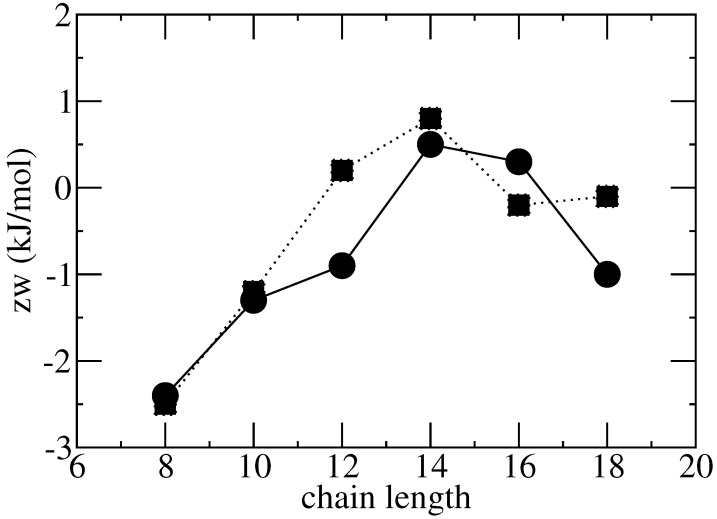
zw versus chain length for menthol–monocarboxylic acids (filled circles and continuous line) and thymol–monocarboxylic acids (filled squares and dotted line).

In the examples reported in Figure 1 and Figure 2, the parameter zw correlates with the length of the chain of the monocarboxylic acids (Figure 3), which may be interpreted as the effect of increasing favorable interactions in the solid compared to the liquid mixture, due to longer apolar chains. Conversely, polar interactions, which are widely regarded to be responsible for DESs, are less and less important as the apolar part of the molecule increases. The interpretation tends to break down at higher lengths of the chain, where the assumption of an equal number of contacts may be inadequate.

Hydrogen bonding has been suggested as a determinant for the behavior of deep eutectic solvents. Thymol, for instance, displays asymmetric hydrogen bonding which energetically favors the mixture compared to pure substances. Therefore, a more negative zw value was expected in comparison with menthol. However, zw includes all contributions averaged and, for the two series of mixtures, the experimental results are similar and, in general, show modest deviations compared to ideal mixtures. The found values of zw, which parallel the similarity of experimental results, also suggest that other contributions, not necessarily enthalpic, might be important and compensate for the expected stronger hydrogen bonding.

### 2.2. Comparison of zw with PC-SAFT Interaction Energy Parameters

Although zw is introduced as a purely enthalpic parameter, it must take into account all deviations from ideality, both enthalpic and entropic. It is, however, expected to be correlated with the corresponding energy parameters in more accurate models, e.g., in the PC-SAFT model.

For the menthol–monocarboxylic acid compounds, there are different energetic parameters: dispersive energy $\Delta u=u_{ij}-\frac{\left(u_{i}+u_{j}\right)}{2}$ and association energy $\Delta \epsilon =\epsilon_{ij}\left(1-k_{ij,eps}\right)-\frac{\left(\epsilon_{i}+\epsilon_{j}\right)}{2}$, according to the definitions given in [14,15,26]. Negative values of $k_{ij,eps}$ are associated with cross-association [28] and Δϵ is therefore a quantity which is positive when cross-association in the liquid is stronger than association in the pure compounds. Another parameter of the model is the number of segments in the chain $m^{seg}$ which also enters the interaction energy through mixing rules and contributes a multiplicative factor to the dispersive energy [15]. The latter parameter is related to the length of the carbon chains for monocarboxylic acids.

In the PC-SAFT model, the solid–liquid equilibrium temperature depends on many parameters, and similar mixtures like thymol–monocarboxylic acids and menthol–monocarboxylic acids, displaying similar behavior, may be described by quite different parameters. For instance, in the just cited example, the association energy is zero for all thymol–monocarboxylic acid mixtures, whereas it is not for menthol–monocarboxylic acid mixtures.

For the menthol–monocarboxylic acid mixtures the Pearson correlation coefficients of zw with the dispersive energy Δu, the association energy Δϵ, and the pairwise interaction term $k_{eps}$ are 0.89, −0.55, and 0.55, respectively.

For the thymol–monocarboxylic acid mixtures, the only energetic parameter is Δu, and the Pearson correlation coefficient of zw with Δu is 0.81.

The Pearson correlation coefficients of zw with the model number of segments in the monocarboxylic acid chain ($m^{seg}$) are 0.70 and 0.69 for the menthol and thymol mixtures, respectively.

**Figure 4:**
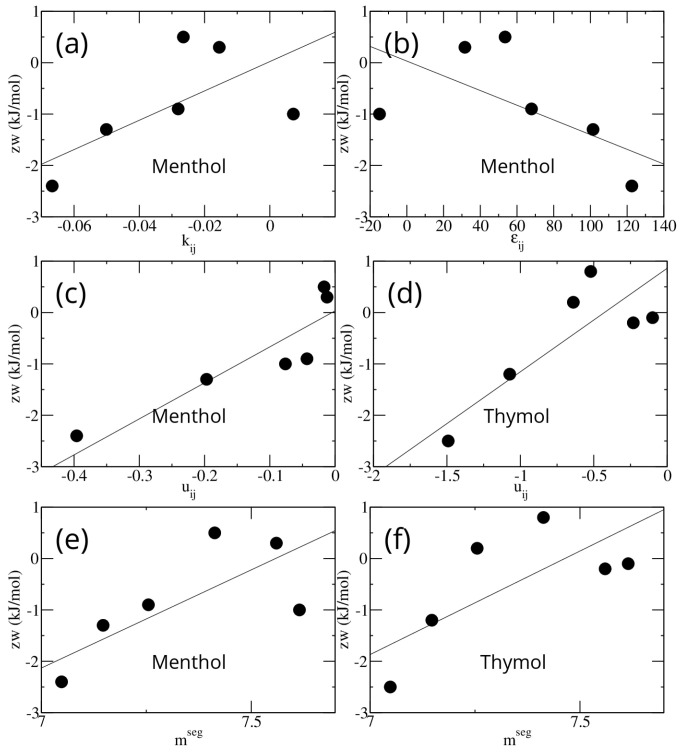
The zw parameters obtained by fitting all experimental data available for eutectic mixtures of menthol and thymol with six different monocarboxylic acids, plotted versus the parameters of the corresponding PC-SAFT models. The continuous line passing through the points is the linear regression line. (**a**) zw versus $k_{ij}$ and (**b**) versus $\epsilon_{ij}$ for menthol. $\epsilon_{ij}$ is zero for thymol. (**c**) zw vs. $u_{ij}$ for menthol and (**d**) for thymol. (**e**) zw versus $m^{seg}$ for menthol and (**f**) for thymol.

The plots of zw versus the energetic parameters of the PC-SAFT model are shown in Figure 4.

These data confirm that the parameter zw is representative of the difference in the interaction between the mixture and the pure compounds. Note that the quantities Δu and Δϵ depend on the parameters of the pure compounds through combination rules, and, for each pair of compounds, pair-specific binary interaction parameters. It is therefore remarkable that the single parameter entering the Bragg–Williams model achieves such a large correlation with the energetic parameters of a much more complex predictive model, such as that based on PC-SAFT.

### 2.3. Test on Selected Type V DES

Most of the binary mixtures considered in the previous subsections had no or modest deviations from ideal behavior. In order to provide more stringent test cases, we selected the four mixtures with the largest differences in melting point with respect to ideal mixtures, from the dataset assembled by Teixeira et al. [29]. For each of the binary mixtures listed in the Supplementary Material of the Teixeira et al. paper, we collected the lowest melting point and the corresponding molar ratio.

Based on the pure compounds’ enthalpy and temperature of melting, the ideal melting temperature was computed at the molar ratio of the lowest experimental melting point. The compounds were then sorted according to the largest difference between the experimental and ideal eutectic melting temperature $\Delta T_{eu}$.

The four mixtures exhibiting the largest deviation from ideal behavior, at the selected molar ratio, and the related references, are as follows:

- Thymol/camphor [30], $\Delta T_{eu}=-102.1K$.
- Thymol/sobrerol [30], $\Delta T_{eu}=-76.7K$.
- Phenol/trioctylphosphine oxide (TOPO) [31], $\Delta T_{eu}=-75.7K$.
- Malonic acid/trioctylphosphine oxide (TOPO) [32], $\Delta T_{eu}=-70.0K$.

**Figure 5:**
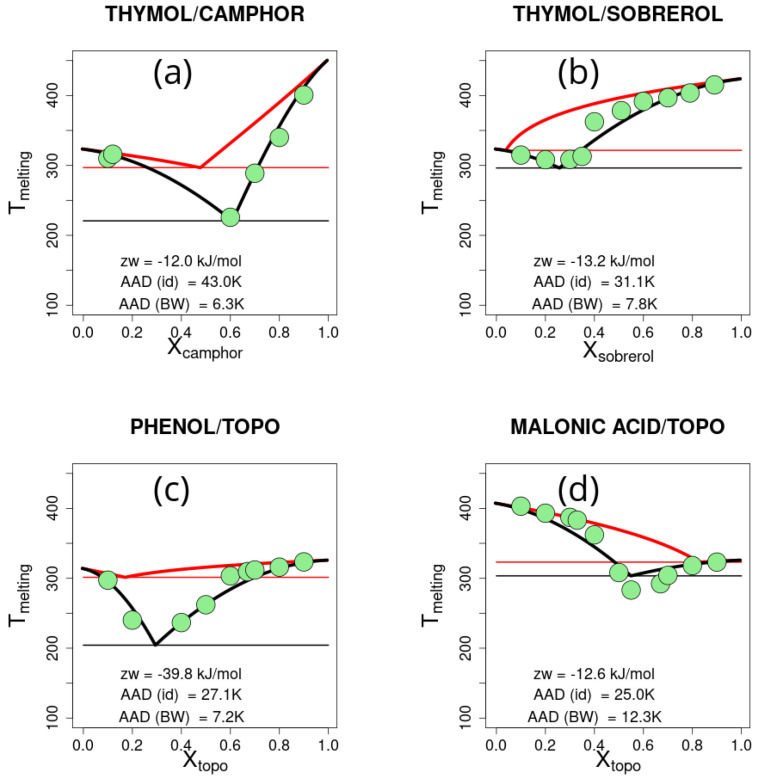
Solid–liquid equilibrium for the selected deep eutectic mixtures: (**a**) thymol/camphor, (**b**) thymol/sobrerol, (**c**) phenol/trioctylphosphine oxide (TOPO), and (**d**) malonic acid/trioctylphosphine oxide (TOPO). Experimental points are show as green filled circles. The solid–liquid equilibrium curves are drawn for ideal mixtures (continuous red line) and the Bragg–Williams model (continuous black line). Horizontal lines are drawn at the lowest melting temperature. The corresponding best-fit Bragg–Williams model zw parameter and the absolute average deviation (AAD) for the ideal (id) and Bragg–Williams (BW) models are reported in the plot.

For each of the selected mixtures, the experimental melting temperatures versus molar ratios were collected and the best-fitting zw parameter was found for the Bragg–Williams model. The results are reported in Figure 5.

Reproducing the solid–liquid equilibrium curves for systems with large deviations from the ideal mixtures is a very stringent test of the Bragg–Williams model. From Figure 5, the quantitative agreement between the experimental points and computed curves may be appreciated. The average AAD in the four plots is 8.40 ± 2.67 K, which largely improves the agreement with respect to the ideal mixture, which shows AAD of 31.57 ± 8.05 K. The zw parameters in the four cases are largely negative, ranging from −12.0 kJ/mol to −39.8 kJ/mol, consistent with the large effects observed.

### 2.4. Error Analysis on zw

In principle, the parameter zw can be obtained from the knowledge of a single molar fraction/melting temperature pair. Indeed, for each experimental point, two zws are found, using the melting temperature and the melting data and molar fraction for each compound. The smaller of the two zws is then chosen, because the larger one would result, for the other compound, in a melting temperature higher than the one the analysis is based on. For instance, in this way, for the terpene and monocarboxylic acid mixtures analyzed above, by choosing the experimental point closer to the 1:1 ratio, to be at the same distance in the molar ratio from both pure constituents, zw parameters are obtained that have a Pearson correlation of 0.88 with those obtained by fitting all points, and their average absolute difference is 0.43 ± 0.38 kJ/mol.

Notwithstanding these results, some important issues must be kept in mind:

(1) The estimate depends on the choice of the experimental point and on the distance of the melting temperature from the ideal solution melting temperature;
(2) As shown in the Section 3, different points are affected by different errors. In particular, points close to the boundaries (i.e., $X_{A}=1$ or $X_{B}=1-X_{A}=1$) are affected by very large errors.

These considerations are illustrated by considering the four type V deep eutectic solvent mixtures analyzed above, which display large changes with respect to the ideal eutectic equilibrium.

**Figure 6:**
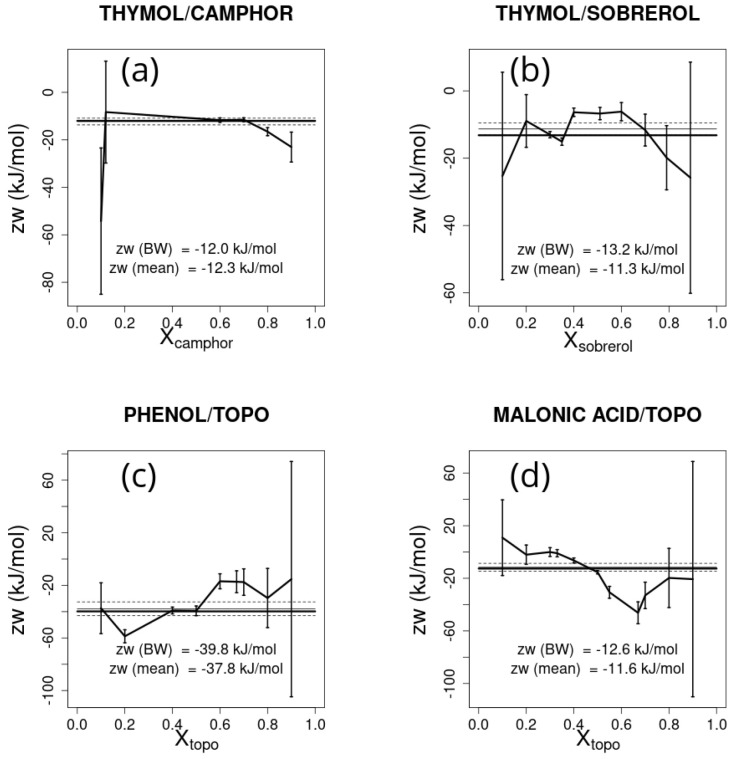
zw and the associated error estimated for each experimental point for the selected deep eutectic solvent mixtures, assuming an error in the temperature of the experimental points of 5 K: (**a**) thymol/camphor, (**b**) thymol/sobrerol, (**c**) phenol/trioctylphosphine oxide (TOPO), and (**d**) malonic acid/trioctylphosphine oxide (TOPO). The reference Bragg–Williams best-fitting zw parameter is shown as a thick horizontal line and the weighted average zw from the experimental points is represented by the thin line bracketed between the two dashed lines representing its associated error.

**Figure 7:**
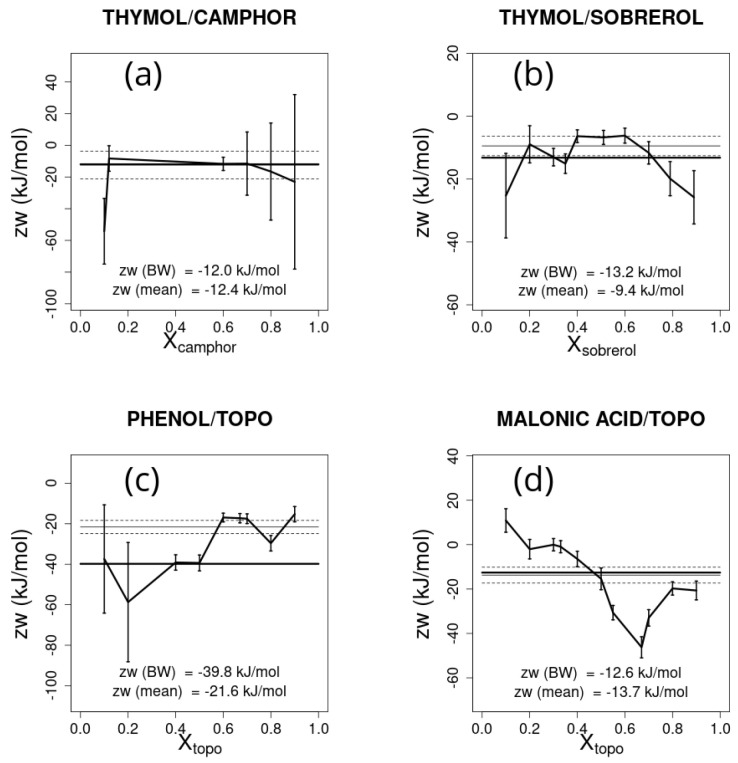
zw and the associated error estimated for each experimental point for the selected deep eutectic solvent mixtures, assuming an error in $\Delta H_{m}^{•}$ of 5 kJ/mol: (**a**) thymol/camphor, (**b**) thymol/sobrerol, (**c**) phenol/trioctylphosphine oxide (TOPO), and (**d**) malonic acid/trioctylphosphine oxide (TOPO). The reference Bragg–Williams best-fitting zw parameter is shown as a thick horizontal line and the weighted average zw from the experimental points is represented by the thin line bracketed between the two dashed lines representing its associated error.

**Figure 8:**
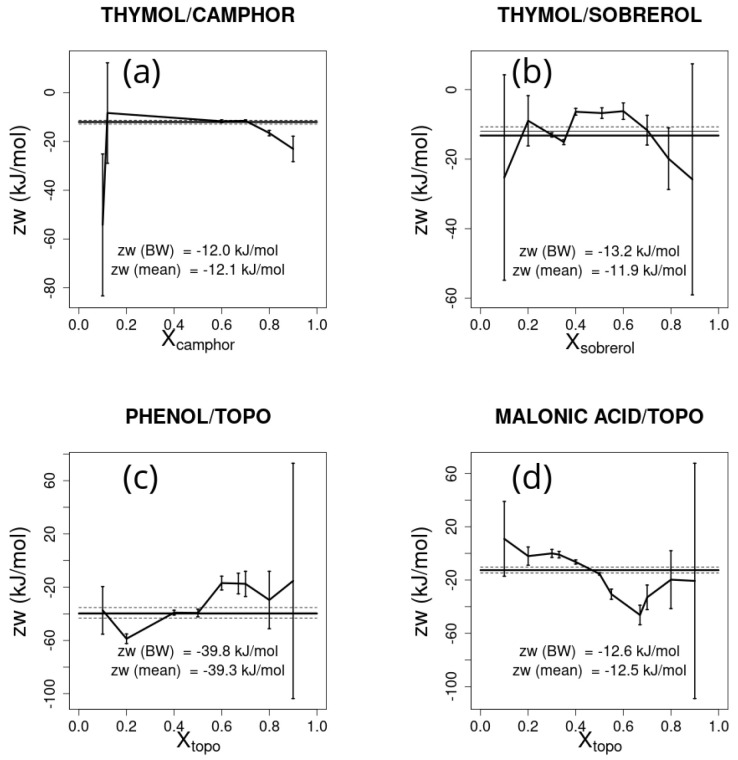
zw and the associated error estimated for each experimental point for the selected deep eutectic solvent mixtures, assuming an error in $T_{m}$ of 5 K: (**a**) thymol/camphor, (**b**) thymol/sobrerol, (**c**) phenol/trioctylphosphine oxide (TOPO), and (**d**) malonic acid/trioctylphosphine oxide (TOPO). The reference Bragg–Williams best-fitting zw parameter is shown as a thick horizontal line and the weighted average zw from the experimental points is represented by the thin line bracketed between the two dashed lines representing its associated error.

The estimate of zw obtained for each molar fraction is reported in Figure 6 together with error bars, assuming an error in the temperature of 5 K. The average zw and the associated error, weighted by the inverse of the square error, is also reported in Figure 6. The large variation of predicted values based on the chosen point is apparent. Whether one or a few points are chosen to estimate the zw parameter, these should be chosen in points possibly far from molar fractions 0 and 1, and where the difference with respect to the ideal eutectic melting temperature is large. The same caveat holds when considering the effect that uncertainties in $\Delta H_{A,m}^{•}$ and $T_{A,m}$ (or those for *B*) have on zw. Here, assuming an uncertainty of 5 kJ/mol in the $\Delta H_{m}^{•}$s and of 5 K in the $T_{m}$s, large errors are also found approaching the ends of the molar fraction range, as shown in Figure 7 and Figure 8, respectively. Note that the effect of errors can lead to large differences in the weighted average of the estimated parameter zw, as shown in Figure 7c.

## 3. Materials and Methods

### 3.1. Ideal Solutions

We provide here the framework to introduce the Bragg–Williams model for the description of the (deep) eutectic properties of liquid mixtures. We consider two substances, *A* and *B*, with the enthalpy of melting (fusion) for the pure substance, e.g., *A*, defined as:

(1) $$\Delta H_{A,m}^{•}=\Delta H_{A,s\to l}$$

Here, the subscripts *s* and *l* stand for solid and liquid, respectively, *m* stands for melting, and quantities with a filled circle superscript refer to pure compounds.

The chemical potentials of the component *A* in the solid and liquid states are:

(2) $$\begin{array}{ccc} \mu_{A,s}^{•} & = & \frac{\partial G_{A,s}^{•}}{\partial n_{A}} \end{array}$$

(3) $$\begin{array}{ccc} \mu_{A,l}^{•} & = & \frac{\partial G_{A,l}^{•}}{\partial n_{A}} \end{array}$$

where $n_{A}$ is the number of moles of *A* and the standard Gibbs free energies for the solid and liquid phases are denoted as $G_{A,s}^{•}$ and $G_{A,l}^{•}$, respectively. For ideal binary solutions characterized by zero enthalpy of mixing, the chemical potential of substance *A* in the liquid phase is the following:

(4) $$\begin{array}{ccc} \mu_{A,l,X_{A}} & = & \mu_{A,l}^{•}+RT\log\left(X_{A}\right) \end{array}$$

where $X_{A}=\frac{n_{A}}{n_{A}+n_{B}}$ is the molar fraction of *A*, *R* is the universal gas constant, and *T* is the absolute temperature.

For a binary solution we will use only the variable $X_{A}$ in the following because $X_{B}=1-X_{A}$.

If the two substances are immiscible in their solid state, at the melting temperature, the chemical potential of the substance in the solid state will be that of the pure substance in the solid state and it will be equal to the chemical potential of the substance in solution at the given composition:

(5) $$\mu_{A,s}^{•}=\mu_{A,l,X_{A}}=\mu_{A,l}^{•}+RT\log\left(X_{A}\right)$$

Thus, for substance *A*:

(6) $$\mu_{A,s}^{•}-\mu_{A,l}^{•}=RT\log\left(X_{A}\right)$$

At the melting point for the pure substance *A*, $\mu_{A,s}^{•}-\mu_{A,l}^{•}=0$. We now consider changes in temperature *T* and molar fraction $X_{A}$ such that the equality $\mu_{A,s}^{•}-\mu_{A,l,X_{A}}=0$ is preserved. We divide first by *T* and consider:

(7) $$d\left(\frac{\mu_{A,l,X_{A}}}{T}\right)-d\left(\frac{\mu_{A,s}^{•}}{T}\right)=\left(\frac{\partial \frac{\mu_{A,l}^{•}}{T}}{\partial T}{|}_{X_{A}}-\frac{\partial \frac{\mu_{A,s}^{•}}{T}}{\partial T}{|}_{X_{A}}\right)dT+R\frac{\partial \log(X_{A})}{\partial X_{A}}{|}_{T}dX_{A}=0$$

(8) $$-\frac{\Delta H_{A,m}^{•}}{T^{2}}dT=-Rd\log\left(X_{A}\right)$$

Integrating from ($X_{A}=1$, $T=T_{A,m}$) to ($X_{A}$, *T*) and assuming $\Delta H_{A,m}^{•}$ changes are negligible, i.e., constant pressure heat capacity $\Delta c_{p}\approx 0$, the lowering of the melting point depends on the solute molar fraction $X_{A}$ and on $\Delta H_{A,m}^{•}$:

(9) $$\frac{1}{T}=\frac{1}{T_{A,m}}-\frac{R\log(X_{A})}{\Delta H_{A,m}^{•}}$$

The solid–liquid equilibrium temperature *T* for the ideal solution is referred to as the ideal solid–liquid equilibrium temperature $T_{id,A}$ for compound *A* and $T_{id}=\max\left(T_{id,A},T_{id,B}\right)$ for the mixture.

### 3.2. Regular Solutions—Bragg–Williams Model

As above, if we assume that the two substances are immiscible in their solid state, the chemical potential of the substance in solution at a given composition includes the entropic term ($RT\log(X_{A})$), as above, but also a possible different interaction enthalpy compared with the pure liquid interactions, say

(10) $$\Delta H=\left(n_{A}+n_{B}\right)H_{l,X_{A}}-n_{A}H_{l,A}^{•}-n_{B}H_{l,B}^{•}$$

The notation $H_{l,X_{A}}$ denotes the molar enthalpy of a mixture of $n_{A}$ and $n_{B}$ moles of *A* and *B*, respectively. The molar enthalpy difference upon mixing ($\Delta H_{A,l}\left(X_{A},T\right)$) in the liquid phase for substance *A* is:

(11) $$\Delta H_{A,l}\left(X_{A},T\right)=\frac{\partial \Delta H}{\partial n_{A}}$$

Under this assumption, the chemical potential for substance *A* (and similarly, for *B*) is:

(12) $$\mu_{A,s}^{•}=\mu_{A,l,X_{A}}=\mu_{A,l}^{•}+RT\log\left(X_{A}\right)+\Delta H_{A,l}\left(X_{A},T\right)$$

Assuming the following:

(1) H≈E, where *E* is the internal energy. This amounts to neglecting the pressure times volume term in the enthalpy *H*;
(2) Each molecule A can make $z_{A}$ contacts with other molecules and each molecule B can make $z_{B}$ contacts with other molecules;
(3) $z_{A}=z_{B}=z$;
(4) Contacts are proportional to mole fractions.

We can express the enthalpy of the solution as:

(13) $$H_{l,n_{A},n_{B}}=\frac{1}{2}n_{A}E_{AA}z\frac{n_{A}}{n_{A}+n_{B}}+n_{A}E_{AB}z\frac{n_{B}}{n_{A}+n_{B}}+\frac{1}{2}n_{B}E_{BB}z\frac{n_{B}}{n_{A}+n_{B}}$$

where $E_{AA}$, $E_{AA}$, and $E_{AB}$ are the interaction energies for each AA, BB, and AB contact, respectively. Similarly, for the pure substances in the liquid states we have:

(14) $$\begin{array}{ccc} n_{A}H_{l,A}^{•} & = & \frac{1}{2}n_{A}zE_{AA} \end{array}$$

(15) $$\begin{array}{ccc} n_{B}H_{l,B}^{•} & = & \frac{1}{2}n_{B}zE_{BB} \end{array}$$

Hence:

(16) $$\begin{array}{ccc} \Delta H & = & H_{l,n_{A},n_{B}}-n_{A}H_{l,A}^{•}-n_{B}H_{l,B}^{•}\;\;\;\;\;\;\;\;\;\;\;\; \end{array}$$

(17) $$\begin{array}{ccc} & = & n_{A}\frac{n_{B}}{n_{A}+n_{B}}zE_{AB}-n_{A}\frac{n_{B}}{n_{A}+n_{B}}z\frac{E_{AA}}{2}-n_{B}\frac{n_{A}}{n_{A}+n_{B}}z\frac{E_{BB}}{2} \end{array}$$

Taking the derivative with respect to $n_{A}$, after straightforward manipulation, we obtain:

(18) $$\Delta H_{A,l}\left(X_{A}\right)={\left(1-X_{A}\right)}^{2}zw$$

where

(19) $$w=E_{AB}-\frac{E_{AA}}{2}-\frac{E_{BB}}{2}$$

is the energy change for breaking half of the AA and BB contacts in the pure liquids to form one AB contact in the mixture.

*w* sets the regime for deviations from ideal solutions. w=0 means an ideal solution, w>0 means that the mixture energy is larger than the energy of pure liquids, and w<0 means that the mixture energy is more favorable than the energy of pure liquids.

Similarly to the treatment for ideal solutions, we can calculate how the melting temperature changes, modifying Equation (7) by adding the term $\Delta H_{A,l}\left(X_{A}\right)={\left(1-X_{A}\right)}^{2}zw$ to the expression for the chemical potential of *A* in the liquid phase:

(20) $$\begin{array}{ccc} d\left(\frac{\mu_{A,l,X_{A}}}{T}\right)-d\left(\frac{\mu_{A,s}^{•}}{T}\right) & = & \\ \left(\frac{\partial \frac{\mu_{A,l}^{•}}{T}}{\partial T}{|}_{X_{A}}-\frac{\partial \frac{\mu_{A,s}^{•}}{T}}{\partial T}{|}_{X_{A}}+\frac{\partial \frac{zw{\left(1-X_{A}\right)}^{2}}{T}}{\partial T}{|}_{X_{A}}\right)dT+\left(R\frac{\partial \log(X_{A})}{\partial X_{A}}{|}_{T}+\frac{1}{T}\frac{\partial zw{\left(1-X_{A}\right)}^{2}}{\partial X_{A}}{|}_{T}\right)dX_{A} & = & 0 \end{array}$$

Equation (20) may be integrated, giving the relation between the solid–liquid equilibrium temperature and the molar fraction:

(21) $$\frac{1}{T}=\frac{1}{T_{A,m}}-\frac{R}{\Delta H_{A,m}^{•}}\log\left(X_{A}\right)-\frac{zw}{\Delta H_{A,m}^{•}}\frac{1}{T}{\left(1-X_{A}\right)}^{2}$$

or

(22) $$T=\frac{1+\frac{zw}{\Delta H_{A,m}^{•}}{\left(1-X_{A}\right)}^{2}}{\frac{1}{T_{A,m}}-\frac{R}{\Delta H_{A,m}^{•}}\log\left(X_{A}\right)}$$

From the latter equation, we can estimate the only free parameter of the model, i.e., zw, with caveats discussed later from each experimental point:

(23) $$zw=\frac{\Delta H_{A,m}^{•}\left(\frac{T}{T_{A,m}}-1\right)-RT\log\left(X_{A}\right)}{{\left(1-X_{A}\right)}^{2}}$$

With the zw parameter, it is then possible to draw the whole phase change curve.

The attractiveness of such a model is that it represents all deviations from ideality in a single parameter that has a direct physical interpretation. It must be clear that there are other models that reproduce better the melting behavior of (deep) eutectic mixtures (and many other properties), even with a limited number of parameters, in comparison to the Bragg–Williams model, but they are in general complex and require many more data for fitting. Therefore, we retain that the model presented here is extremely useful for a simple yet quantitative interpretation of the lowering of the melting temperatures in deep eutectic mixtures. Some examples will be presented in the Results section.

### 3.3. Other Related Models

We note that the present derivation for the Bragg–Williams model is analogous to that of Lee [21], who did not make explicit the number of contacts, but rather used a single parameter $\rho_{0}$, which is the same as zw in this work. It is also worth noticing that the starting point of the Bragg–Williams model described here, i.e., the excess enthalpy $\Delta H_{A,l}\left(X_{A},T\right)$, is virtually the same as for the Margules activity model [33] and the regular solution theory by Hildebrand [34]. In both models, the equation is obtained following a similar but different line of thought, i.e., activity coefficients are assumed to be smooth with respect to molar fractions and simple expressions for them are hypothesized. The equation for $\Delta H_{A,l}\left(X_{A},T\right)$, identical to the one in the Bragg–Williams model, is obtained by truncating the activity coefficient expression at the first term of the expansion in powers of molar fraction [33].

### 3.4. Error Analysis

The model has only one parameter (zw) to describe the solid–liquid equilibrium curve and therefore errors in this parameter due to the limited potency of the model are expected. We consider that molar fractions are known with good accuracy and we consider the errors in temperature (ΔT) important, because of experimental limitations, but also because of the intrinsic inaccuracy of the model, affecting the estimate of zw. From the propagation of errors, the expected error in zw is:

(24) $$\Delta zw=\left|\frac{\frac{\Delta H_{A,m}^{•}}{T_{A,m}}-R\log\left(X_{A}\right)}{{\left(1-X_{A}\right)}^{2}}\right|\Delta T$$

Note that since the expression at the denominator tends to 0 as $X_{A}$ tends to 1, errors in the estimated equilibrium temperature become extremely large close to both ends of the molar fraction range.

For most compounds, $\Delta H_{A,m}^{•}$ and $T_{A,m}$ which enter the expression for zw are known from the literature with great accuracy; however, this could also be a source of error. Similar to the above, by propagation of errors on $\Delta H_{A,m}^{•}$ and $T_{A,m}$, we obtain:

(25) $$\Delta zw=\left|\frac{\frac{T}{T_{A,m}}-1}{{\left(1-X_{A}\right)}^{2}}\right|\Delta \left(\Delta H_{A,m}^{•}\right)$$

and

(26) $$\Delta zw=\left|\frac{\frac{\Delta H_{A,m}^{•}T}{T_{A,m}^{2}}}{{\left(1-X_{A}\right)}^{2}}\right|\Delta T_{A,m}$$

Also, here, the term ${\left(1-X_{A}\right)}^{2}$ and, at the other end, considering *B*, ${\left(1-X_{B}\right)}^{2}$, in the denominator lead to a large error close to the boundaries of the molar fraction range.

A last issue to consider is how the solid–liquid equilibrium curve depends in the Bragg–Williams model on the uncertainty of the parameter zw. From Equation (22), upon substitution of $\frac{1}{T_{A,m}}-\frac{R}{\Delta H_{A,m}^{•}\log\left(X_{A}\right)}$ with $\frac{1}{T_{id,A}}$, we find:

(27) $$\Delta T=\left|\frac{{\left(1-X_{A}\right)}^{2}}{\Delta H_{A,m}^{•}}T_{id,A}\left(X_{A}\right)\right|\Delta zw$$

From the latter equation, it can be seen that the dependence of the solid–liquid equilibrium curve does not display a singular point but it can be large, depending on the ratio of $\frac{\Delta zw}{\Delta H_{A,m}^{•}}$. It therefore appears advantageous to fit zw on all points available to reduce the overall error in the solid–liquid equilibrium temperature *T*.

### 3.5. Thermodynamic Data for Pure Compounds

All the treatments developed above and the resulting Equations (21) and (23) require knowledge of the enthalpy and temperature of melting of pure compounds. For type V DESs [27], a list of such quantities has been assembled by Coutinho and collaborators [29]. The data for all the pure compounds used in the present work are reported in Table 1.

## 4. Conclusions

Many theoretical models have been proposed to describe, among other properties, the solid–liquid equilibrium of mixtures. Apparently, the Bragg–Williams model, developed to describe metal alloys and later applied to many other systems, has not been explored so far to describe the solid–liquid equilibrium of eutectic mixtures.

Here, we have presented the Bragg–Williams model describing mixtures through a single parameter, i.e., the product of the number of contacts of each molecule times the difference of the contact energy between unlike and like molecules. This single parameter is directly related to the depression or increase in the melting temperature with respect to ideal solutions.

Compared to the accuracy typically achieved with other (more complex) models in which several parameters must be fitted, the description of the solid–liquid equilibrium of mixtures by the Bragg–Williams model is somewhat less accurate. The average absolute deviation (AAD), with respect to experiments, of the calculated melting temperatures for menthol and thymol mixtures with six monocarboxylic acids is 1.38 ± 0.81 K, which is larger but comparable to the accuracy achieved with PC-SAFT modeling (AAD 1.15 K). The energy parameter fitted to the experimental data correlates well with analogous energetic parameters of the PC-SAFT model, which provides a confirmation for the soundness of the Bragg–Williams model.

Whereas other thermodynamic models require a large number of points to fit model parameters, the Bragg–Williams model presented here uses a single energetic parameter zw that can be estimated, in principle, even from a single experimental point chosen, e.g., close to the molar fraction 0.5, but in practice, even from a few experimental points far from the molar fractions 0 and 1, and where the melting temperature is different from the ideal solution melting temperature.

The dependence of the calculated zw parameter on the chosen experimental points highlights the limitations of the approach and therefore, fitting more points far from the 0 or 1 molar fraction is necessary.

Extensions of the method could include modeling different numbers of possible contacts for each compound or/and describing ternary mixtures.

In the first possible extension, different valency parameters $z_{A}$ and $z_{B}$ would be introduced, slightly changing the equations and leading to similar formulae in terms of the $z_{A}w$ and $z_{B}w$ parameters, analogous to the single zw. However, the dependence of the melting temperature on the molar fraction would be more complicated, because *z* does not simplify when taking the probability of the contacts, and the relevant quantities are the number of moles times the number of possible contacts, rather than just the number of moles.

The second possible extension would involve three components with two independent mole fractions. In a ternary Bragg–Williams model, without the specifications of any other restriction, the simplicity of the approach presented here would be lost, making it necessary to introduce energetic parameters for the AB, AC, and BC contacts.

The limitations of the binary Bragg–Williams model are apparent; the assumption of the same number of contacts for the two molecular species restricts, in principle, its applicability to mixtures of similar-sized compounds. The freedom in fitting the parameter could, however, reduce the problem. A second restriction is that it considers only very simple phase diagrams. It has been repeatedly shown that phase diagrams may be much more complex than considered here [27], and also for mixtures involving molecules addressed in the present work like stearic acid [35]. The model does not apply to the latter situations.

Besides possible developments and additional parameters, this work supports the idea that, beyond the zeroth-order treatment of eutectic mixtures by the ideal solution model, the Bragg–Williams model might be a useful first-order reference model for such important physicochemical systems. It provides useful thermodynamic information on the system requiring minimal input data. It is also worth noticing that the model is ready for use for any experimental group without extensive prior training in statistical mechanics or machine learning. Some example scripts in R (v.4.2.1) are provided as Supplementary Materials.

## Figures and Tables

**Table 1:** Thermodynamic data for pure compounds used in this work. $T_{m}$ is the temperature of melting and $\Delta H_{m}$ is the enthalpy of melting [29].

Compound	$T_{m}$ (K)	$\Delta H_{m}$ (kJ/mol)
Menthol	315.7	12.89
Thymol	323.5	19.65
Caprylic acid	289.5	21.38
Capric acid	304.8	27.5
Lauric acid	317.5	37.83
Myristic acid	327.03	41.29
Palmitic acid	336.8	51.02
Stearic acid	343.7	61.36
Trioctylphosphine oxide	325.9	58.02
Camphor	450.4	5.28
Sobrerol	423.9	34.81
Phenol	314.15	11.8635
Malonic acid	407.46	23.1

## Data Availability

The data used in this manuscript have been obtained from the cited references.

## Supplementary Materials

The following supporting information can be downloaded at: https://www.mdpi.com/article/10.3390/ijms26030997/s1.

## Abbreviations

The following abbreviations are used in this manuscript:

AAD	Average absolute deviation
DES	Deep eutectic solvents
COSMO-RS	COnductor-like Screening MOdel for Real Solvents
PC-SAFT	Perturbed chain statistical associating fluid theory
TAG	Triacylglycerol
TOPO	Trioctylphosphine oxide
